# Effects of Ventromedial Hypothalamic Nucleus (VMN) Aromatase Gene Knockdown on VMN Glycogen Metabolism and Glucoregulatory Neurotransmission

**DOI:** 10.3390/biology12020242

**Published:** 2023-02-03

**Authors:** Karen P. Briski, A. S. M. Hasan Mahmood, Md. Main Uddin, Mostafa M. H. Ibrahim, Khaggeswar Bheemanapally

**Affiliations:** School of Basic Pharmaceutical and Toxicological Sciences, College of Pharmacy, University of Louisiana Monroe, Monroe, LA 71201, USA

**Keywords:** aromatase, ventromedial hypothalamic nucleus, insulin-induced hypoglycemia, neuronal nitric oxide synthase, glutamate decarboxylase, gene knockdown

## Abstract

**Simple Summary:**

The brain is a target for steroid hormones derived from the circulation or synthesized locally. Central estrogen receptors control neural functions that preserve whole-body energy stability and optimize blood levels of glucose, a principal metabolic fuel. Glucose insufficiency owing to insulin-induced hypoglycemia, an unremitting complication of type I diabetes mellitus management, can injure or destroy brain neurons. It is critical to understand how neuroestradiol may affect neural circuitries that initiate physiological and behavioral responses that rectify hypoglycemia. Current studies investigated whether estradiol generated by the enzyme aromatase acts within the key component of central nervous system regulation of glucose balance, the ventromedial hypothalamic nucleus, to regulate metabolic-sensitive neurochemical signaling. Here, state-of-the-art reagents for manipulation of in vivo aromatase gene expression were stereotactically delivered to that nucleus to investigate whether this enzyme protein is essential for optimal insulin-induced hypoglycemia-associated patterns of local neurotransmitter synthesis, involving microdissection-harvesting of pure glucose-stimulatory and glucose-inhibitory neuron cell samples and plasma glucose counter-regulatory hormone profiles. Results provide novel evidence that hypoglycemic adjustments in ventromedial nucleus neuroestradiol synthesis vary over the rostro-caudal length of that structure and that aromatase activity is required increased glucose-stimulatory and for decreased glucose-inhibitory transmission at distinct levels of this nucleus.

**Abstract:**

The enzyme aromatase is expressed at high levels in the ventromedial hypothalamic nucleus (VMN), a principal component of the brain gluco-regulatory network. Current research utilized selective gene knockdown tools to investigate the premise that VMN neuroestradiol controls glucostasis. Intra-VMN aromatase siRNA administration decreased baseline aromatase protein expression and tissue estradiol concentrations and either reversed or attenuated the hypoglycemic regulation of these profiles in a VMN segment-specific manner. Aromatase gene repression down-regulated protein biomarkers for gluco-stimulatory (nitric oxide; NO) and -inhibitory (gamma-aminobutyric acid; GABA) neurochemical transmitters. Insulin-induced hypoglycemia (IIH) up- or down-regulated neuronal nitric oxide synthase (nNOS) and glutamate decarboxylase_65/67_ (GAD), respectively, throughout the VMN. Interestingly, IIH caused divergent changes in tissue aromatase and estradiol levels in rostral (diminished) versus middle and caudal (elevated) VMN. Aromatase knockdown prevented hypoglycemic nNOS augmentation in VMN middle and caudal segments, but abolished the GAD inhibitory response to IIH throughout this nucleus. VMN nitrergic and GABAergic neurons monitor stimulus-specific glycogen breakdown. Here, glycogen synthase (GS) and phosphorylase brain- (GPbb; AMP-sensitive) and muscle- (GPmm; noradrenergic –responsive) type isoform responses to aromatase siRNA were evaluated. Aromatase repression reduced GPbb and GPmm content in euglycemic controls and prevented hypoglycemic regulation of GPmm but not GPbb expression while reversing glycogen accumulation. Aromatase siRNA elevated baseline glucagon and corticosterone secretion and abolished hypoglycemic hyperglucagonemia and hypercorticosteronemia. Outcomes document the involvement of VMN neuroestradiol signaling in brain control of glucose homeostasis. Aromatase regulation of VMN gluco-regulatory signaling of hypoglycemia-associated energy imbalance may entail, in part, control of GP variant-mediated glycogen disassembly.

## 1. Introduction

Estradiol acts within the brain to regulate a broad contingent of critical functions, such as reproductive behavior and hormone secretion, energy balance, and memory and learning. This control is achieved through concerted signaling by classical nuclear, e.g., estrogen receptor-alpha (ERα) and estrogen receptor-beta (ERβ) and membrane, e.g., G-protein-coupled ER (GPER1/GPR30) ERs [1,2]. Brain ERs are activated by ligands synthesized in the ovary as well as locally. Neuroestradiol is produced in neural tissue by aromatase enzyme-catalyzed conversion of testosterone. Aromatase mRNA and protein expression and enzyme activity profiles vary between neural structures, with elevated levels found in distinct, interconnected structures including the medial amygdala, medial preoptic area, ventromedial hypothalamic nucleus (VMN), and bed nucleus of the *stria terminalis* [3,4,5,6,7,8,9,10]. Aromatase activity is critical for male socio-sexual behavior [11] and female sexual behavior and reproductive neuroendocrine function [12]. There is an evolving consensus that prompt estradiol effects on behavior may involve, to some extent, non-transcriptional ER mechanisms initiated by swift aromatase-facilitated changes in local tissue neuroestradiol levels [13]. Neuroestradiol is reported to regulate several vital behavioral activities under VMN control, including sex-specific reproductive performance [14].

The brain requires an outsized percentage of total energy intake to preserve essential nerve cell functions and is dependent upon glucose as its primary energy substrate. Insulin-induced hypoglycemia (IIH) is an unalleviated drawback of compulsory exacting pharmacotherapeutic control of type 1 or type 2 diabetes mellitus [15,16]. Possible adverse effects of inadequate glucose delivery to the brain caused by IIH involve neurological dysfunction and damage to or destruction of susceptible neurons. When neuro-glucopenia occurs, the brain takes action by triggering counter-active autonomic, neuroendocrine, and behavioral activities that increase glycemic profiles. The ventromedial hypothalamic nucleus (VMN) controls several vital physiological and behavioral functions, such as arousal, maternal, sexual, aggressive, and defensive behaviors, metabolic homeostasis, autonomic outflow, and thermogenesis [17,18,19,20,21]. The VMN fulfills the critical function of assimilation of nutrient, endocrine, and neurotransmitter metabolic cues that govern glucostasis [22,23]. Specialized VMN metabolic-sensory neurons generate a continuous readout of cellular energy status by intensifying (‘fuel-inhibited’) or diminishing (‘fuel-excited’) synaptic firing when ambient energy substrate levels decline [24,25,26]. Optimal counter-regulatory endocrine and gluconeogenic functions are driven by sensory indicators of neuro-energetic instability in the medio-basal hypothalamus (MBH), which encompasses the VMN and other distinctive nuclei [27,28]. Neurochemical effectors of hypothalamic energy imbalance include the amino acid transmitter γ-aminobutyric acid (GABA), which blunts hypoglycemic augmentation of glucagon and adrenomedullary epinephrine [29], as well as the gas nitric oxide (NO), a labile lipid-permeable free radical that augments counter-regulatory hormone release [30,31]. VMN nitrergic and γ-aminobutyric acid GABA-ergic neurons express the hypoglycemia-sensitive energy gauge 5′-AMP-activated protein kinase (AMPK) [32]. GABA and NO signals originate within the VMN, as recent single-cell multiplex qPCR studies show that VMN neurons labeled in situ as immunoreactive-positive for the biomarker glutamate decarboxylase_65/67_ (GAD) or neuronal nitric oxide synthase (nNOS) contain mRNA that encodes both GAD molecular weight variants [33] or nNOS [Briski, personal observation], respectively. VMN expression profiles of nNOS and GAD are subject to sex-dimorphic control by estrogen receptor ER-alpha (ERα) and ER-beta (ERβ) during insulin-induced hypoglycemia (IIH) [34]. Recent pharmacological studies in our laboratory, in which the aromatase inhibitor letrozole was infused into the lateral ventricle, yielded novel proof that forebrain neuroestradiol regulates VMN nNOS and GAD protein expression in distinct rostro-caudal segments of that structure during euglycemia and neuro-glucopenia [35]. Current research builds upon that work through the application of genetic tools for site-specific selective aromatase gene knockdown to address the original premise that neuroestradiol may control nitrergic and/or GABAergic transmission in specific VMN locations and that such an influence aligns with aromatase siRNA effects on counter-regulatory endocrine function. 

Astrocytes maintain the branched polysaccharide glycogen as the preeminent metabolic fuel depot in the brain [36]. In these glia, a significant percentage of glucose acquired from the blood passes through the glycogen shunt ahead of glycolytic pathway conversion to the oxidizable energy fuel L-lactate [37]. Glycogen metabolism is controlled by antagonistic glycogen synthase (GS) and glycogen phosphorylase (GP) enzyme actions, which correspondingly promote glycogen assembly or deconstruction. GP-mediated brain glycogenolysis increases in response to an energy supply/demand imbalance, which occurs, for example, during sleep deprivation, hypoglycemia, or seizure [38,39], for the purpose of generating L-lactate for transfer to neurons [36,40]. Multiple GP isoforms are synthesized in the brain, including muscle- (GPmm) and brain- (GPbb) variants, which differ with cell type and regulation by phosphorylation versus AMP [41]. Astrocytes contain both GPmm and -bb, whereas GPbb is also present, albeit at lesser levels, in neurons. Phosphorylation causes complete (GPmm) versus partial (GPbb) activation of these isoforms, while GPbb is more sensitive to AMP activation than is GPmm and requires AMP binding for optimal enzyme Km and function. GPmm and GPbb reportedly control noradrenergic or glucoprivic induction of glycogenolysis in vitro, respectively [42]. Evidence for up-regulated nNOS expression after intra-VMN administration of the non-selective GP antagonist 1,4-dideoxy-1,4-imino-d-arabinitol (DAB) infers that reductions in glycogen-derived substrate fuel volume may be construed as an indication of energy shortage by VMN gluco-regulatory neurons [43]. Work performed here utilized uHPLC-electrospray ionization mass spectrometry (LC-ESI-MS) techniques previously optimized for analysis of small-volume brain tissue glycogen content [44] to address the corollary hypothesis that VMN neuroestradiol controls stimulus-specific GP isoform regulation of glycogen mass and disassembly during eu- and/or hypoglycemia.

## 2. Materials and Methods

### 2.1. Animals

Adult male Sprague Dawley rats (350–400 gm *bw*) were co-housed (2–3 per cage) under a 14 h light/10 h dark cycle (lights on at 05.00 h). Animals had unrestricted access to standard laboratory chow (Harlan Teklad LM-485; Harlan Industries, Madison, WI, USA) and tap water and were handled daily prior to experimentation. Study protocols were approved in advance by the ULM Institutional Animal Care and Use Committee, and were in compliance with the NIH Guide for the Care and Use of Laboratory Animals, 8th Edition.

### 2.2. Experimental Design

Individual animals, e.g., the experimental unit, were assigned randomly to four treatment groups, which consisted of *n* = 4 rats per group. Surgeries were carried out in the College of Pharmacy Vivarium, at the dedicated surgical suite located adjacent to housing quarters. Animal health and welfare status were appraised by investigators and Vivarium management on a daily basis over the course of the study. On study day 1, rats were anaesthetized by subcutaneous (*sc*) injection of ketamine/xylazine (0.1 mL/100 g *bw*; 90 mg ketamine:10 mg xylazine/mL; Henry Schein Inc., Melville, NY, USA) prior to bilateral stereotactic administration (as described by Mahmood et al. in 2019) of Scramble (SCR) siRNA (500 pmol; Accell™ Control Pool Non-Targeting siRNA, prod. no. D-001910-10-20; Horizon Discovery Group, Cambridge, UK; groups 1 and 2), or aromatase siRNA (500 pmol; Accell™ siRNA Rat cyp19a1, set of 4; prod. no. A-097560-13/14/15/16-0005; Horizon Disc. [Suzuki et al., 2010]) into the VMN, at predetermined coordinates: −2.50 mm posterior to *bregma*; 0.6 mm lateral to midline; 9.0 mm below skull surface. Upon completion of surgery, rats were treated by injection of ketoprofen (1 mg/mL/kg *bw sc*; Covetrus, Dublin, OH, USA) and enrofloxacin (10 mg/0.1 mL IM; Covetrus) and topical application of bupivacaine ointment (0.25%; Hospira Inc., Lake Forest, IL, USA) to closed incisions. After post-surgical surveillance, animals were settled into single-occupancy cages. On study day 7, at 09.00 h, rats were given a *sc* injection of either sterile insulin diluent (V; Eli Lilly & Company, Indianapolis, IN, USA; groups 1 and 3) or neutral protamine Hagedorn insulin (INS; 10 U/kg *bw*; groups 2 and 4) [34]. No rats were eliminated from the study owing to health complications. Animals were sacrificed on day 7 at 10 a.m. by microwave fixation (4.5 kW energy; 1.45 s exposure; In Vivo Microwave Fixation System, prod. no. 50037; Stoelting Co., Wood Dale, IL, USA) to maximize brain tissue glycogen preservation [32]. Brains were snap-frozen by rapid immersion in liquid nitrogen-cooled isopentane and saved at −80 °C. Plasma samples were kept at −20 °C.

### 2.3. Neural Tissue Sample Procurement

Each animal’s forebrain was cut into consecutive 100 μm-thick fresh-frozen sections along the longitudinal length of the VMN, e.g., from −1.80 to −3.3 mm posterior to the *bregma*. A calibrated round stainless-steel hollow micropunch tool (0.5 mm internal diameter; prod. no. 57401; Stoelting Co., Kiel, WI, USA) was used to bilaterally dissect VMN tissue from sections collected through rostral (−1.8 to −2.3 mm), middle (−2.3 to −2.8 mm), and caudal (−2.8 to −3.3 mm) VMN segments for either (1) Western blotting (2 × 100 μm-thick sections per segment) or (2) uHPLC-electrospray ionization-mass spectrometric (LC-ESI-MS) analyses (3 × 100 μm-thick sections per segment). Bilateral micropunched tissue samples were also obtained from adjacent hypothalamic structures that are implicated in glucose homeostasis, e.g., hypothalamic paraventricular (−0.7 mm to −2.3 mm), dorsomedial (−2.0 mm to −3.8 mm), and arcuate (−1.8 mm to −3.2 mm) nuclei and lateral hypothalamic area (−2.3 mm to −3.8 mm), in order to carry out immunoblot analysis to assess whether AROM siRNA delivery to the VMN affects AROM protein expression in those neighboring sites.

### 2.4. Western Blot Analysis of VMN Target Proteins

Tissue samples from each subject were collected separately for the rostral, middle, and caudal VMN regions. For each treatment group, tissue lysate aliquots from each individual animal were pooled to create quadruplicate sample pools for each target protein. Stain-Free Imaging Technology, involving total protein measurement, was used as the loading control. Sample pools were electrophoresed in Bio-Rad TGX 10% stain-free gels (Bio-Rad, Hercules, CA, USA); after separation, gels were activated by UV light (1 min) in a Bio-Rad ChemiDoc MP Imaging System to measure total protein in each lane [32,34,35]. Proteins were transferred to 0.45-μm PVDF-Plus membranes (prod. no. 121639; Data Support Co., Panorama City, CA, USA), which were processed for washes and antibody incubations by FreedomRocker™ Blotbot^®^ automation (Next Advance, Inc., Troy, NY, USA). Non-specific immunoreagent binding was minimized by pretreating membranes with Tris-buffer saline, pH 7.4, 10 mM tris hydrochloride, and 50 mM sodium chloride (TBS) supplemented with 0.1% Tween-20 and 2% bovine serum albumin. Membranes were incubated (36–42 h, 4 °C) with rabbit primary polyclonal antisera raised against aromatase (1:2000; prod. no. NB100-1596, Novus Biologicals, Littleton, CO, USA), GAD (1:10,000; prod. no. ABN904; Millipore Sigma Burlington, MA, USA), nNOS (1:2000; prod. no. NBP1-39681; Novus Biol.), GPbb (1:2000; prod. no. NBP1-32799; Novus Biol.), GPmm (1:2000; prod. No. NBP2-16689, Novus Biol.), or GS (prod. no. NBP1-3893S; Novus Biol.). Membranes were then exposed to goat anti-rabbit horseradish peroxidase-labeled secondary antibodies (1:5000; prod. no. NEF812001EA; PerkinElmer, Waltham, MA, USA) prior to incubation with maximum-sensitivity SuperSignal WestFemto chemiluminescent substrate (prod. no. 34096; Thermo Fisher Scientific, Rockford, IL, USA). The chemiluminescence optical density (O.D.) value measured for each target protein band was normalized to the total protein in that lane using ChemiDoc MP Image Lab™ 6.0.0 software (Bio-Rad; https://www.bio-rad.com/en-us/applications-technologies/stain-free-imaging-technology?ID=NZ0G1815 (accessed on 27 January 2023). Bio-Rad Stain-Free Gels contain a proprietary trihalo compound incorporated directly into the gel chemistry; this compound lacks inherent fluorescence but renders in-gel proteins fluorescent upon UV photoactivation and thus measurable by O.D. Software sums all individual protein optical densities in a single lane and relates that total protein O.D. value to the target protein O.D. in the same lane, thereby deriving a normalized O.D. value. This advanced Western blot normalization method distinctly reduces data variability through enhanced measurement accuracy and precision [45,46]. Each Western blot analysis employed precision plus protein molecular weight dual color standards (prod. no. 161-0374, Bio-Rad). Our figures depict this, as Y axis labels denote mean normalized O.D. measures. The formula used for normalization is the ratio of specific target protein O.D./total in-lane protein O.D.

### 2.5. LC-ESI-MS Analysis of VMN Glycogen Concentrations

Tissue from separate VMN segments was processed by heat-denaturation, homogenization, and ultrasonification. Lysate supernatants (10 μL) were hydrolyzed by 2 h of incubation with 0.5 mg/mL amyloglucosidase (10 μL) and 0.1 M sodium acetate, pH 5.0 (10 μL), then sequentially heated (100 °C; 5 min) and cooled to room temperature. Tissue glycogen levels were analyzed in a ThermoFisherScientific (TFS; Waltham, MA, USA) Vanquish UHPLC + System using ThermoScientific™ Dionex™ Chromeleon™ 7 Chromatography Data System software [44]. Column (35 °C) and autosampler (10 °C) temperatures were held constant. The auto-sampler needle was washed with 10% (*v*/*v*) methanol (10 s). Hydrolyzed and non-hydrolyzed samples were derivatized with 100 μL of 0.5 M 1-phenyl-3-methyl-5-pyrazolone (PMP) reagent supplemented with 0.3 M NaOH. After acidification with 400 μL of 0.75% formic acid and extraction with chloroform, supernatant aliquots (400 μL) were vacuum-concentrated, frozen at −80 °C, and lyophilized. Lyophilization products were diluted to 1.0 mL with 10 mM ammonium acetate, bath-sonicated (30 s), then centrifuged. Supernatant aliquots (250 μL) were transferred to 350 μL inserts secured in 2 mL Surestop vials in an autosampler tray. D-(+)-Glucose-PMP was resolved using the Shodex™ Asahipak™ NH2P-40 3E column (Shodex, NY, USA) with a mobile phase (75:25 *v*/*v*) and acetonitrile:10 mM ammonium acetate (0.2 mL/min). D-(+)-Glucose-PMP ion chromatograms were obtained at *m*/*z* 510.2 to yield area-under-the-curve data. Critical LC-ESI-MS parameters, such as sheath gas pressure (SGP; 25 psig), auxiliary gas pressure (AGP; 4.6 psig), sweep gas pressure (SWGP; 0.5 psig), vaporizer temperature (VT; 150 °C), ion transfer tube temperature (ITT; 150 °C), source voltage (−2000 V), foreline pressure (1.76 Torr; auto-set by instrument- and variable), source gas (nitrogen; Genius NM32LA 110 V, 10–6520; Peak Scientific, Inchinnan, Scotland), and mass peak area detection algorithm (ICIS/Genesis) were each maintained at an optimum [44]. A TFS NanoDrop One microvolume UV-Vis spectrophotometer (840-274200) (ThermoFisherScientific, Waltham, MA, USA) was used to measure sample protein content; tissue glycogen concentrations were expressed as ug/mg protein.

### 2.6. LC-ESI-MS Analysis of VMN Estradiol Concentrations

Rostral, middle, and caudal VMN tissues were pooled separately in 100 μL of ultrapure water, then saved at −80 °C. Thawed sample aliquots were vortexed (30 s), homogenized, and centrifuged to yield clear supernatant aliquots for combination with the internal standard (IS) in water. Oasis^®^ MCX (3 cc/60 mg, Waters) cartridges were used for steroid solid phase extraction (SPE) [47]. Cartridges were eluted with methanol (MeOH; 2 mL; VWR, Radnor, PA), water (2 mL), and formic acid (FA; VWR; 2% *v*/*v*, 2 mL) prior to acetonitrile (ACN; VWR) elution of estrogens. Residues were dried, lyophilized, and derivatized with 2-fluoro-1-methylpyridiniump-toluenesulfonate (TCI, Portland, OR, USA). A chromatography system consisting of a TFS UHPLC Vanquish binary pump (VFP10A01/121345), a Vanquish auto-sampler (VFA10A02/121345), and a temperature-controlled Vanquish UHPLC + column compartment (VHC10A02/121345) was paired with a TFS single quad ISQ EC mass spectrometer (ISQECLC/121345). ThermoScientific™ Dionex™ Chromeleon™ 7 Chromatography Data System software (7200.0300/121345; TFS) was utilized for mass spectrometric analysis. A Kinetex 2.6 μm PS C18 100 Å (100 mm L × 2.1 mm ID) P/No. 00D-4780-AN column (Phenomenex, Torrance, CA, USA) was used at 0.2 mL/min flow. The auto-sampler needle was washed (10 s) with 10% (*v*/*v*) MeOH. Mobile phases A and B consisted of 1.77 mM FA and ACN, respectively; isocratic phase flow of ACN (75%) and FA (25%) was maintained for 4 min. A FMP-E-2 (*m*/*z* 364.2) calibration curve was established with FMP-ethinyl estradiol as IS, as this derivative exhibited a highly stable mass spectrometric response (*m*/*z* 388.2) in standard and brain samples. VMN tissue estradiol concentrations were reported as pg/mg protein.

### 2.7. Plasma Glucose and Counter-Regulatory Hormone Analyses

Plasma glucose levels were measured using an ACCU-CHECK Aviva-plus glucometer (Roche Diagnostic Corporation, Indianapolis, IN, USA) [34]. Plasma corticosterone (cat# ADI-900-097; Enzo Life Sciences, Inc., Farmingdale, NY, USA) and glucagon (cat# EZGLU-30K; EMD Millipore, Billerica, MA, USA) concentrations were analyzed using commercial ELISA kit reagents [34].

### 2.8. Statistical Analyses

Mean normalized VMN segmental protein O.D., estradiol, and glycogen measures, and plasma glucose, glucagon, and corticosterone concentrations were evaluated by two-way analysis of variance and the Student-Newman-Keuls *post-hoc* test, using GraphPad Prism software. Differences of *p* < 0.05 were interpreted as indicative of statistical significance. For each figure, statistical differences in mean values of distinct pairs of treatment groups are indicated as follows: * *p* < 0.05; ** *p* < 0.01; *** *p* < 0.001; **** *p* < 0.0001.

## 3. Results

**Figure 1:**
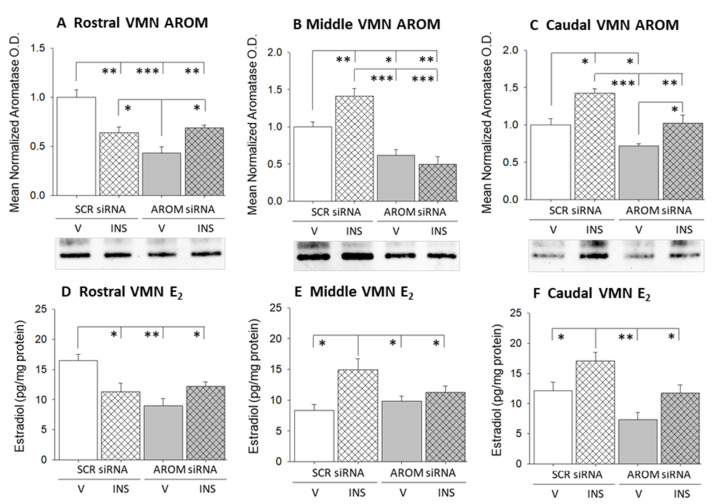
Effects of Intra-Ventromedial Hypothalamic Nucleus (VMN) Aromatase siRNA Administration on Tissue Aromatase Protein and Estradiol Content in Euglycemic and Hypoglycemic Male Rats. Bilateral, micropunched samples from rostral, middle, and caudal VMN were obtained from groups of scramble (SCR) or aromatase (AROM) siRNA-pretreated male rats 1 h following subcutaneous (sc) injection of vehicle (V) or neutral protamine Hagedorn insulin (INS; 10 U/kg *bw*). (**A**–**C**) show mean normalized rostral, middle, and caudal VMN aromatase protein optical density (O.D.) measures + S.E.M., respectively, for SCR siRNA/V (solid white bar; *n* = 4), SCR siRNA/INS (diagonal-striped, white bar; *n* = 4), and AROM siRNA/V (solid gray bar; *n* = 4), AROM siRNA/INS (diagonal-striped gray bar; *n* = 4) treatment groups. (**D**–**F**) depict mean rostral, middle, and caudal VMN tissue estradiol concentrations + S.E.M. after V or INS injection to SCR versus aromatase siRNA-pretreated animals. * *p* < 0.05; ** *p* < 0.01; *** *p* < 0.001.

Figure 1 depicts the effects of bilateral aromatase siRNA administration on tissue aromatase (AROM) protein content (Figure 1A–C) and estradiol concentrations (Figure 1D–F) in the rostral, middle, and caudal segments of the VMN. Data show that this targeted drug delivery decreased AROM protein expression in rostral (Figure 1A; F_(3,8)_ = 16.24; *p* = 0.0009), middle (Figure 1B; F_(3,8)_ = 22.79; *p* = 0.0003), and caudal (Figure 1C; F_(3,8)_ = 14.70; *p* = 0.0013) VMN segments [AROM siRNA/V (solid gray bars) versus SCR siRNA/V (solid white bars)]. Representative full, uncropped immunoblots of rostral, middle, and caudal VMN AROM proteins are shown in Supplementary Figures S1A, S2A, and S3A, respectively. INS injection stimulated (rostral VMN) or diminished (middle and caudal VMN) AROM protein profiles, according to region [SCR siRNA/INS (diagonal-striped white bars) versus SCR siRNA/V (solid white bars)]. AROM gene knockdown prevented hypoglycemic effects on this protein in the rostral and middle VMN but did not avert INS-induced stimulation of AROM protein levels in the caudal VMN segment [AROM siRNA/INS (diagonal-striped, gray bars) versus SCR siRNA/INS (diagonal-striped white bars)]. AROM siRNA treatment caused a significant diminution of tissue estradiol concentrations in rostral (Figure 1D; F_(3,12)_ = 7.50; *p* = 0.003) and caudal (Figure 1F; F_(3,12)_ = 8.60; *p* = 0.0026) regions of the VMN. Effects of IIH on VMN estradiol levels were segment-specific, as this steroid profile declined in the rostral VMN (F_(3,12)_ = 7.50; *p* = 0.003), but was elevated in the middle (Figure 1E; F_(3,12)_ = 4.89; *p* = 0.019) and caudal VMN (F_(3,12)_ = 8.60; *p* = 0.003). AROM gene knockdown prevented hypoglycemia-associated changes in tissue estradiol concentrations in each VMN segment.

**Figure 2:**
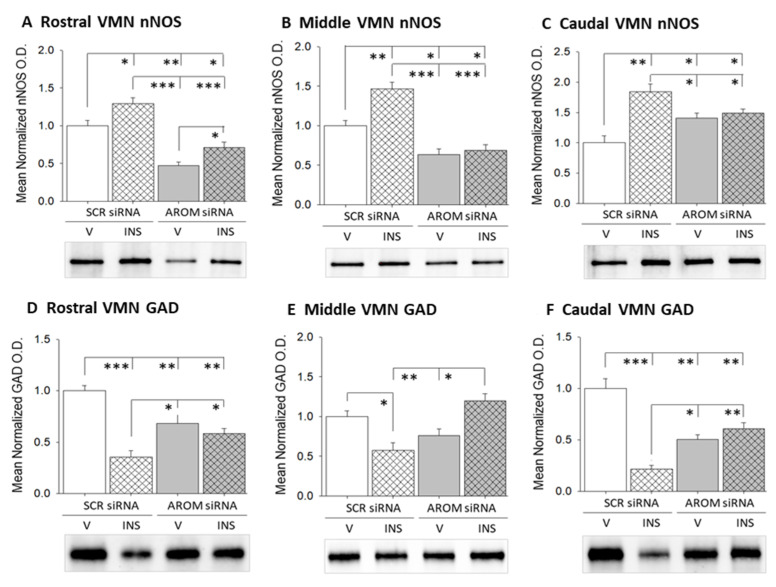
Impact of VMN aromatase gene knockdown on rostral, middle, and caudal VMN gluco-stimulatory and gluco-inhibitory transmitter marker protein expression in V-versus INS-injected rats. Data show mean normalized neuronal nitric oxide synthase (nNOS) ((**A**), rostral VMN; (**B**), middle VMN; (**C**), caudal VMN) and glutamate decarboxylase_65/67_ (GAD) ((**D**), rostral VMN; (**E**), middle VMN, (**F**), caudal VMN) protein O.D. values + S.E.M. for SCR siRNA/V (solid white bars; *n* = 4), SCR siRNA/INS (diagonal-striped white bars; *n* = 4), AROM siRNA/V (solid gray bars; *n* = 4), AROM siRNA/INS (diagonal-striped gray bars; *n* = 4) treatment groups. * *p* < 0.05; ** *p* < 0.01; *** *p* < 0.001.

**Figure 3:**
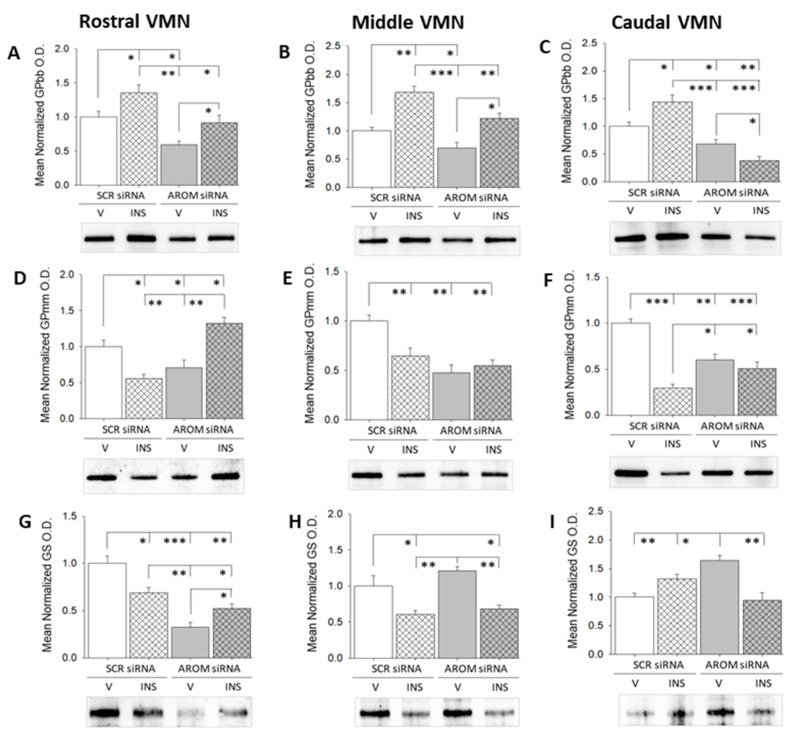
Eu- and hypoglycemic patterns of VMN glycogen metabolic enzyme protein expression in SCR versus aromatase siRNA-pretreated male rats. Data depict mean normalized glycogen phosphorylase (GP)-brain type (GPbb; (**A**–**C**)), GP-muscle type (GPmm; (**D**–**F**)), and glycogen synthase (GS; (**G**–**I**)) protein O.D. values + S.E.M. for SCR siRNA/V (solid white bars; *n* = 4), SCR siRNA/INS (diagonal-striped white bars; *n* = 4), AROM siRNA/V (solid gray bars; *n* = 4), AROM siRNA/INS (diagonal-striped gray bars; *n* = 4) treatment groups. * *p* < 0.05; ** *p* < 0.01; *** *p* < 0.001.

Figure 2 shows patterns of nNOS (Figure 2A–C) or GAD (Figure 2D–F) protein expression in the rostral, middle, and caudal VMN of rats pretreated by intra-VMN administration of SCR or AROM siRNA prior to *sc* V versus INS injection. Supplementary Figures S1–S3 depict full-length immunoblots of rostral, middle, and caudal VMN nNOS (Figure 1B, Figure 2B, and Figure 3B) or GAD (Figure 1C, Figure 2C, and Figure 3C) protein expression in each treatment group. Euglycemic animals treated by intra-VMN administration of the AROM siRNA showed decreased [rostral VMN (Figure 2A; F_(3,8)_ = 25.91; *p* = 0.0002); middle VMN (Figure 2B; F_(3,8)_ = 28.48; *p* = 0.0001), or increased [caudal VMN (Figure 2C; F_(3,8)_ = 12.08; *p* = 0.002] tissue nNOS protein content, according to VMN segment [AROM siRNA/V (solid gray bars) versus SCR/V (solid white bars)]. Hypoglycemia increased nNOS expression in each region investigated; this stimulatory effect was averted by VMN AROM gene knockdown in the middle and caudal VMN segments. Rostral (Figure 2D; F_(3,8)_ = 17.55; *p* = 0.0007), middle (Figure 2E; F_(3,8)_ = 10.29; *p* = 0.004), and caudal VMN (Figure 2F; F_(3,8)_ = 25.62; *p* = 0.0002) GAD protein content was suppressed by AROM siRNA administration. INS injection inhibited this profile in each rostro-caudal segment. Hypoglycemic inhibition of GAD expression was prevented by VMN AROM gene repression.

Patterns of glycogen metabolic enzyme protein expression in distinct rostro-caudal VMN segments after SCR siRNA/V, SCR siRNA/INS, AROM siRNA/V, or AROM siRNA/INS treatments are shown in Figure 3 Data show that VMN AROM gene knockdown diminished baseline expression profiles of the AMP-sensitive GP variant GPbb in rostral (Figure 3A; F_(3,8)_ = 10.94; *p* = 0.0033), middle (Figure 3B; F_(3,8)_ = 15.84; *p* = 0.0005), and caudal (Figure 3C; F_(2,8)_ = 24.96; *p* = 0.0002) regions of the VMN. Hypoglycemia up-regulated this protein profile in each rostro-caudal segment of this nucleus; however, this stimulatory response was averted by AROM gene knockdown only in the caudal VMN. Supplementary figures show uncropped rostral (Figure 1D), middle (Figure 2D), and caudal (Figure 3D) VMN GPbb protein Western blots. Repression of AROM also down-regulated levels of the norepinephrine-sensitive variant GPmm throughout the VMN [rostral segment (Figure 3D; F_(2,8)_ = 15.98; *p* = 0.001), middle segment (Figure 3E; F_(2,8)_ = 11.14; *p* = 0.003), and caudal segment (Figure 3F; F_(2,8)_ = 26.53; *p =* 0.0002). INS injection suppressed GPmm expression over the rostro-caudal length of the VMN. This inhibitory response was abolished by AROM siRNA pretreatment. Full-length rostral (Figure 1E), middle (Figure 2E), and caudal (Figure 3E) VMN GPmm immunoblots are shown in Supplementary Figures S1–S3. Tissue GS levels were down-regulated [rostral VMN (Figure 3G; F_(2,8)_ = 23.14; *p* = 0.0003), unaffected by [middle VMN (Figure 3H; F_(2,8)_ = 10.17; *p* = 0.004)], or increased [caudal VMN (Figure 3I; F_(2,8)_ = 10.87; *p* = 0.003)] by VMN AROM gene knockdown. Supplementary figures show uncropped rostral (Figure 1F), middle (Figure 2F), and caudal (Figure 3F) VMN GPbb protein Western blots. Hypoglycemia suppressed GS expression in the rostral and middle segments; AROM siRNA pretreatment prevented this decline in the rostral but not the middle VMN. INS did not modify caudal VMN-GS tissue levels in SCR-pretreated animals but suppressed this protein after AROM siRNA pretreatment.

**Figure 4:**
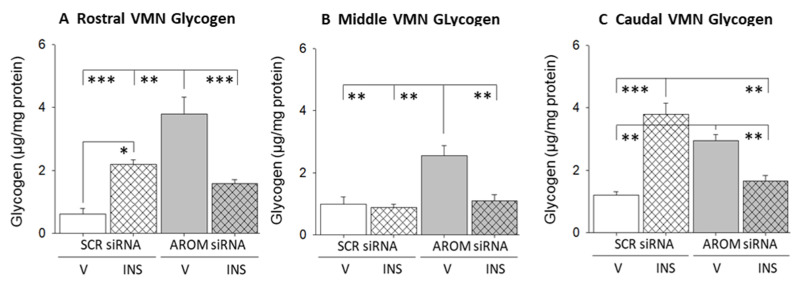
Effects of VMN aromatase gene knockdown on tissue glycogen concentrations in eu- and hypoglycemic male rats. Data depict mean rostral (**A**), middle (**B**), and caudal (**C**) VMN tissue glycogen concentrations ± S.E.M. for groups of V- or INS-injected, SCR versus AROM siRNA-pretreated animals (*n* = 4 per treatment group). * *p* < 0.05; ** *p* < 0.01; *** *p* < 0.001.

Data in Figure 4 depict the effects of VMN AROM gene knockdown on rostral, middle, and caudal VMN tissue glycogen accumulation. Full-length immunoblots illustrating rostral, middle, and caudal VMN GAD protein in individual treatment groups are depicted in Supplementary Figures S1C, S2C, and S3C, respectively. Control V-injected euglycemic animals pretreated with aromatase siRNA showed significant augmentation of mean tissue glycogen concentrations in rostral (Figure 4A; F_(3,12)_ = 17.50; *p* = 0.0001), middle (Figure 4B; F_(3,12)_ = 8.05; *p* = 0.0033), and caudal (Figure 4C; F_(3,12)_ = 14.01; *p* = 0.0003) VMN segments. INS injection elevated rostral and caudal VMN glycogen content, whereas middle VMN glycogen mass was refractory to this experimental treatment. Hypoglycemia-associated augmentation of glycogen accumulation was averted by aromatase gene knockdown in a region-specific manner.

**Figure 5:**
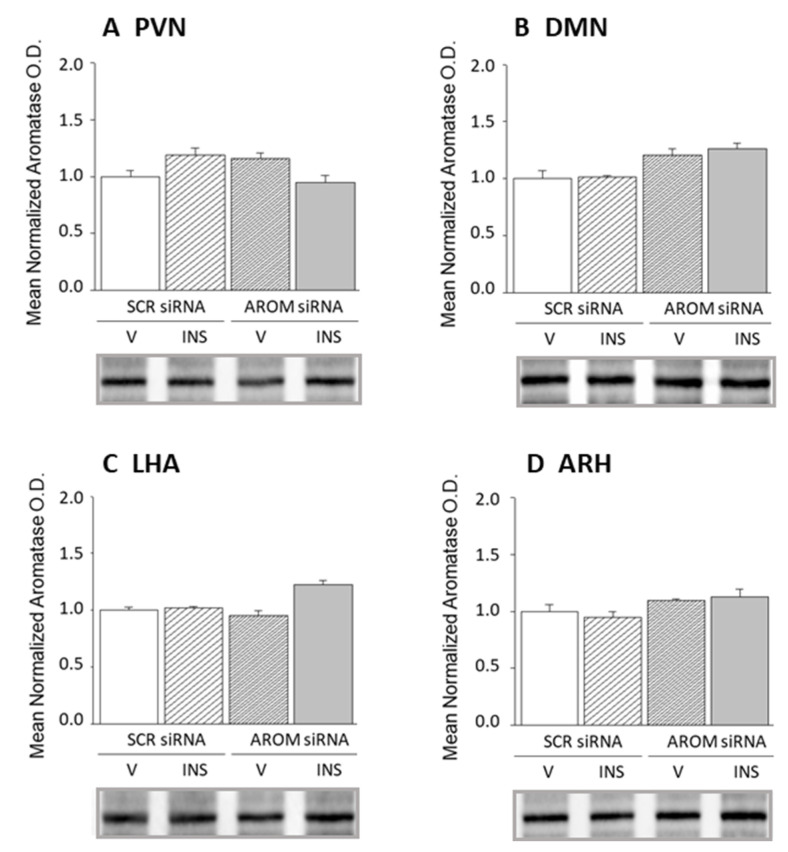
Effects of aromatase siRNA delivery to the VMN on aromatase protein expression in the paraventricular (PVN), dorsomedial (DMN), and arcuate (ARH) hypothalamic nucleus and lateral hypothalamic area (LHA). Data show mean normalized PVN (**A**), DMN (**B**), LHA (**C**), or ARH (**D**) AROM protein O.D. values + S.E.M. after administration of stereotactic SCR or AROM siRNA delivery to the VMN ahead of *sc* V or INS injection (*n* = 4/treatment group).

Effects of AROM siRNA administration to the VMN on tissue AROM protein levels in adjacent hypothalamic metabolic loci, e.g., PVH, DMN, LHA, or ARH, are illustrated in Figure 5 and Supplementary Figure S4. Data show that this treatment did not significantly alter AROM expression profiles in any of these sites. These findings bolster the supposition that demonstrable AROM knockdown effects on VMN target protein levels likely reflect VMN nerve cell responses to altered AROM gene expression within the confines of the VMN.

**Figure 6:**
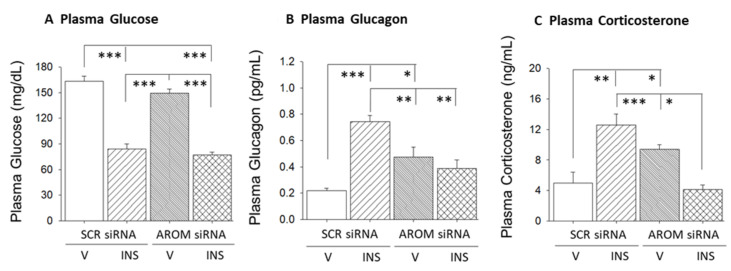
VMN aromatase regulation of plasma glucose and counterregulatory hormone profiles. Data illustrate effects of intra-VMN SCR or AROM siRNA administration on mean circulating glucose (**A**), glucagon (**B**), and corticosterone (**C**) concentrations ± S.E.M. for V- or INS-injected, SCR versus AROM siRNA-pretreated animals (*n* = 4 per treatment group). * *p* < 0.05; ** *p* < 0.01; *** *p* < 0.001.

Data in Figure 6 show the effects of VMN siAROM RNA administration on circulating glucose and counter-regulatory hormone levels. Data show that plasma glucose levels were unaffected by VMN AROM gene knockdown (Figure 6A). INS injection significantly decreased circulating glucose; this response was unaffected by aromatase siRNA pretreatment (F_(3,12)_ = 69.04; *p* < 0.0001). Plasma glucagon concentrations were elevated in siRNA-pretreated, V-injected animals (Figure 6B; F_(3,12)_ = 14.53; *p* = 0.0003). Hypoglycemia elevates glucagon secretion relative to the euglycemic control range. Aromatase siRNA pretreatment normalized patterns of glucagon secretion in INS-injected animals. VMN aromatase gene knockdown also augmented baseline corticosterone secretion (Figure 6C; F_(3,12)_ = 12.85; *p =* 0.0005). INS-associated augmentation of corticosterone secretion was averted by aromatase siRNA pretreatment.

## 4. Discussion

Current research utilized self-delivery siRNA technology in conjunction with combinatory immunocytochemistry/laser-catapult microdissection, Western blotting, and high-sensitivity uHPLC-electrospray ionization-mass spectrometry to address the notion that VMN aromatase regulates local glucose-regulatory transmission and glycogen metabolism alongside systemic counter-regulatory hormone profiles. Data show that VMN AROM gene knockdown down-regulated baseline nNOS and GAD expression throughout this neural structure and averted or attenuated hypoglycemic regulation of these protein profiles, according to the rostro-caudal segment. Hypoglycemia caused divergent changes in VMN GPbb (increased) and GPmm (decreased) protein levels, responses that were refractory to (GPbb) or reversed by (GPmm) aromatase siRNA pretreatment. AROM siRNA augmented glycogen accumulation over the longitudinal length of the VMN; IIH-associated changes in glycogen content, which were reversible by AROM gene repression, were only observed in rostral or caudal segments. Segment-based molecular analysis of the VMN reveals here that IIH regulation of transmitter marker and glycogen metabolic enzyme protein profiles and glycogen amassment will possibly depend upon down- (rostral VMN) or up- (middle and caudal VMN) regulated neuroestradiol signaling. VMN AROM gene knockdown elevated glucagon and corticosterone secretion in euglycemic animals and prevented hypoglycemic stimulation of the release of these hormones. Outcomes verify that neuroestradiol signaling within the VMN shapes glucose-regulatory neurochemical and glycogen metabolic responses to hypoglycemia and is required for optimal counter-regulatory outflow during this metabolic challenge.

Brain nuclear and membrane ERs are activated by estradiol that is either delivered to the brain via the circulation or produced locally by aromatase. Acute changes in tissue estradiol concentrations owing to altered neuroestradiol synthesis presumably elicit effects, in part, through non-genomic mechanisms. Further research is warranted to determine if neuroestradiol controls VMN glucose-regulatory function by action on GPER alone or by signaling through GPER along with membrane ERα and/or ERβ. It would be informative to examine the role of systemic estradiol in VMN AROM expression and activity patterns during eu- versus hypoglycemia, and to ascertain if optimum neuroestradiol control of glucose-regulation necessitates coincident ER activation by gonadal estradiol. As VMN nitrergic and GABAergic neurons express GPER as well as ERα and ERβ [48], it is plausible that both neuron populations may be direct substrates for neuroestradiol action. The possibility that neuroestradiol may also control nNOS and/or GAD expression by acting on upstream neurons that innervate these metabolic-sensory neurons cannot be overlooked at present. Neuroestradiol is also apt to directly regulate VMN astrocyte GP isoform expression, as these glia express GPER, ERα, and ERβ [49].

Western blot analysis of rostral, middle, and caudal micropunch-dissected VMN tissue showed that AROM siRNA administration to that nucleus caused significant diminution of AROM protein expression over the length of the VMN (summarized in Table 1). Current research highlights the efficacy of high-performance LC-ESI-MS for high neuroanatomical resolution quantification of brain tissue estradiol concentrations. Application of this methodology here showed that VMN AROM knockdown caused region-specific reductions in estradiol levels as tissue steroid profiles decreased in the rostral and caudal VMN regions, but not the middle segment. These findings imply that a substantial proportion of total estradiol concentrations associated with distinctive VMN regions consists of neuroestradiol. Divergent AROM siRNA effects on aromatase protein versus estradiol concentrations in the middle VMN suggest that suppression of neuroestradiol production in this location may elicit adaptively up-regulated estradiol uptake from the circulation, or alternatively, that AROM enzyme activity in this site is intensified in response to decreased protein expression levels.

Hypoglycemia-correlated patterns of estradiol signaling in the VMN likely reflect neuroestradiol output as tissue AROM protein and estradiol levels undergo parallel adjustments, and moreover, aromatase siRNA averted hypoglycemic regulation of tissue estradiol concentrations in each VMN segment. Notably, hypoglycemia suppressed both AROM and estradiol levels in the rostral VMN, yet elevated these profiles in other VMN regions. The latter data concur with previous reports of divergent, segment-contingent effects of INS therapy on VMN AROM protein expression in the male [48]. Equivalent effects of down- versus up-regulated regional AROM expression on glucose-regulatory transmitter marker or glycogen metabolic enzyme proteins may reflect net equivalence of tissue estradiol levels across segments due to hypoglycemia, or alternatively, regional differences in ERs and/or post-receptor signal transduction mechanisms involved in local neuroestradiol control of those targets. AROM gene knockdown prevented hypoglycemic aromatase expression patterns in rostral and middle VMN segments, but not in the caudal VMN. It could thus be presumed that the magnitude of decrements in this protein profile in all but the caudal region of the VMN may be a determinant of sensitivity to hypoglycemia.

VMN AROM knockdown was found here to down- (rostral and middle VMN) or up- (caudal VMN) regulate nNOS expression in euglycemic animals, according to rostro-caudal segment. These results characterize regional VMN nitrergic neuron subpopulations that are alternatively governed by a stimulatory or inhibitory neuroestradiol tone. At the same time, baseline VMN GAD protein content was reduced throughout the VMN following AROM siRNA treatment, indicative of uniform positive neuroestradiol control of VMN GABAergic transmission. AROM gene knockdown attenuated hypoglycemic up-regulation of middle and caudal, but not rostral VMN nNOS protein. These results likely document the loss (rostral VMN) or gain (caudal VMN) of neuroestradiol regulation of this protein profile as a consequence of a switch from a state of metabolic stability to imbalance. Current research is unique in denoting differential aromatase regulation of baseline or hypoglycemia-associated NO signaling by neuroanatomically defined subpopulations of VMN nitrergic neurons. Yet, AROM gene repression abolished hypoglycemic diminution of VMN GAD content over the length of the VMN, which infers that GABA neurons exhibit homogeneous responsiveness to neuroestradiol during IIH. Current outcomes point to neuroestradiol action as critical for optimal glucose-inhibitory as well as glucose-stimulatory metabolic transmitter reactivity to IIH.

VMN AROM knockdown suppressed baseline GPbb and GPmm expression throughout this hypothalamic nucleus, outcomes that correlated with elevated tissue glycogen concentrations. These outcomes provide novel proof that neuroestradiol acts within the VMN to regulate local glycogen metabolism. Here, hypoglycemia caused contrary adjustments in GP variant protein profiles, as GPbb and GPmm levels were respectively amplified or diminished. These results infer that neuro-glucopenia likely promotes VMN glycogen disassembly by the glucoprivic-sensitive isoform GPbb, which would conceivably expedite glucosyl monomer release under circumstances of substrate fuel deficiency. Evidence here for hypoglycemia-associated augmentation of rostral and caudal VMN glycogen levels supports the view that, in these sites, GPmm activity is an important determinant of net glycogen mass and that the proportionate glycogen mass that can be deconstructed by GPmm likely exceeds that dismantled by GPbb. Preliminary observations of relatively higher GPmm versus GPbb protein concentrations in the VMN support the latter premise [Briski, personal communication]. Here, AROM siRNA administration prevented hypoglycemic up-regulation of GPbb expression in the caudal VMN only but abolished hypoglycemic suppression of GPmm content in each VMN segment. These findings, along with observations of regional differences in astrocyte GS protein expression profiles in eu- and hypoglycemic rats after AROM knockdown, infer that neuroestradiol may impose segment-specific control of astrocyte glycogen synthesis under conditions of energy sufficiency or deficiency, as well as energy-sensitive GP variant-mediated disassembly during IIH. Data show that AROM gene knockdown prevented glycogen accumulation during hypoglycemia, which suggests that neuroestradiol signaling in the VMN impedes glycogen breakdown by the norepinephrine-sensitive GP variant during IIH. There remains a need to identify the regulatory cues that elevate rostral and middle VMN GPbb expression during glucose insufficiency.

Results show that VMN AROM knockdown did not affect plasma glucose profiles in eu- or hypoglycemic animals. However, rats treated with AROM siRNA exhibited elevated baseline glucagon and corticosterone secretion, which infers that neuroestradiol action on VMN substrates suppresses gluco-stimulatory signaling. Concurrent inhibition of GAD profiles over the rostro-caudal length of the VMN by AROM knockdown raises the prospect that this treatment may augment counter-regulatory hormone outflow, in part, by repressing glucose-inhibitory GABA signaling. Moreover, up-regulated caudal VMN nitrergic transmission may also contribute to heightened glucagon and corticosterone release in AROM siRNA-treated rats. IIH stimulation of these counter-regulatory hormone profiles was averted by VMN aromatase gene knockdown, resulting in outcomes that correlate with prevention of up-regulated middle and caudal VMN nitrergic signaling and deterred down-regulation of GABA transmission over the longitudinal axis of the VMN. One or both of these neurotransmitters may mediate neuroestradiol control of counter-regulatory hormone responses to IIH.

The identity of the VMN cell type(s) that synthesizes neuroestradiol remains to be ascertained. Recent in vitro work shows that primary hypothalamic astrocyte cell cultures contain aromatase protein [49], but further studies are needed to determine if VMN astrocytes produce de novo estradiol in vivo. Hippocampal astrocytes exhibit augmented aromatase expression in response to tissue ischemia [50]; here, it remains to be determined whether VMN astrocytes are capable of adjusting neuroestradiol output in response to regulatory cues of metabolic imbalance. Distinct forebrain neuron populations are evidently able to synthesize neuroestradiol, as indicated by evidence for aromatase expression in immortalized cell lines derived from preoptic and hypothalamic KiSS1/mestatin neurons [51] and in GABAergic neurons located in the quail brain bed nucleus stria terminalis and medial preoptic nucleus, but not the VMN [52]. In the latter study, aromatase immunoreactivity was localized to non-GABA neurons in the VMN, indicating that neuronal neuroestradiol is generated in that nucleus in the non-mammalian species examined there. A technical consideration relevant to current work, that remains unanswered at present, concerns whether aromatase siRNA administration in vivo has equivalent efficacy in suppressing gene expression in neurons versus neuroglia.

In summary, results document a parallel diminution of VMN tissue AROM protein expression and estradiol content caused by intra-VMN aromatase siRNA administration. Outcomes provide evidence for VMN neuroestradiol regulation of basal and hypoglycemic patterns of gluco-stimulatory nitrergic and gluco-inhibitory GABAergic signaling. Data implicate AROM in VMN segment-specific glycogen amassment and in stimulus-specific disassembly of this substrate fuel reserve during hypoglycemia. Current data, moreover, provide novel proof of VMN AROM involvement in neural regulation of baseline and hypoglycemic patterns of counter-regulatory hormone secretion.

## 5. Conclusions

Emerging recognition that VMN neuroestradiol has physiological relevance to neural control of glucose homeostasis will undoubtedly motivate efforts to characterize the transmitter, endocrine, and/or nutrient cues that shape local aromatase activity and thereby affect the volume, i.e., strength of this regulatory signal within distinct rostro-caudal segments of the VMN. There also remains a pressing need to determine if aromatase-expressing VMN cell types exhibit sex-specific receptivity to those cues and, if so, whether sex-dimorphic patterns of neuroestradiol production occur under conditions of glucose sufficiency and/or deficiency. Investigation of post-receptor mechanisms, by which alterations in magnitude of local estradiol release affect target nerve cell input to the central nervous system glucostatic circuitry, is also warranted. The VMN directs a wide, diverse array of critical behaviors and organ system functions in addition to energy homeostasis; it remains to be determined if local neuroestradiol signaling acts as a volume transmitter to synchronize adjustments in these various activities in response to specific physiological or pharmacological stimuli.

Brain astrocytes maintain glycogen as a substitute source of metabolic fuel for neurons in the event of an energy imbalance. Glycogen disassembly is triggered when glucose or blood supply to the brain is diminished or when neurological function is intensified. Astrocyte communication of the eminent loss of this energy reserve is important for neurons, both on an individual and collective basis, to enact adjustments such as diminution or reallocation of energy use, utilization of other non-glucose substrate fuels, and body-wide actions that restore the glycogen depot. More research is warranted to provide insight on the molecular mechanisms of neuroestradiol regulation of astrocyte glycogen metabolism, knowledge that can be leveraged to advance the development of therapeutic strategies for neuro-protective amplification of brain glycogen levels in each sex.

## Figures and Tables

**Table 1:** Summary of Ventromedial Hypothalamic Nucleus (VMN) Aromatase Gene Knockdown Effects on Glucose-Regulatory Transmitter Marker and Astrocyte Glycogen Metabolic Enzyme Protein Expression in Rostral, Middle, versus Caudal VMN.

	Rostral VMN				Middle VMN				Caudal VMN			
	SCR ^a^	siRNA	AROM ^b^	siRNA	SCR	siRNA	AROM	siRNA	SCR	siRNA	AROM	siRNA
	V ^c^	INS ^d^	V	INS	V	INS	V	INS	V	INS	V	INS
AROM	-	↓ ^e^	↓	↑ ^f^	-	↑	↓	N.C. ^g^	-	↑	↓	↑
Estradiol	-	↓	↓	N.C	-	↑	N.C.	N.C.	-	↑	N.C.	N.C.
nNOS ^h^	-	↑	↓	↑	-	↑	↓	N.C.	-	↑	↑	N.C.
GAD ^i^	-	↓	↓	N.C.	-	↓	N.C.	↑	-	↓	↓	N.C.
GPbb ^j^	-	↑	↓	↑	-	↑	↓	↑	-	↑	↓	↓
GPmm ^k^	-	↓	N.C.	↑	-	↓	↓	N.C.	-	↓	↓	N.C.
GS ^l^	-	↓	↓	↑	-	↓	N.C.	↓	-	N.C	↑	↓
Glycogen	-	↑	↑	↓	-	N.C.	↑	↓	-	↑	↑	↓

^a^ scramble (control); ^b^ aromatase; ^c^ sterile diluent subcutaneous (*sc*); ^d^ 10.0 U neutral protamine Hagedorn insulin/kg *bw sc*; ^e^ relative change versus SCR siRNA/V; ^f^ relative change versus AROM siRNA/V; ^g^ no change relative to V; ^h^ neuronal nitric oxide synthase; ^i^ glutamate decarboxylase_65/67_; ^j^ glycogen phosphorylase-brain type; ^k^ glycogen phosphorylase-muscle type; ^l^ glycogen synthase.

## Data Availability

All data generated during and/or analyzed during the current study are available from the corresponding author on reasonable request.

## Supplementary Materials

The following supporting information can be downloaded at: https://www.mdpi.com/article/10.3390/biology12020242/s1, Figure S1: Representative Rostral VMN Target Protein Full-Length Western Blots; Figure S2: Representative Middle VMN Target Protein Full-Length Western Blots; Figure S3: Representative Caudal VMN Target Protein Full-Length Western Blots; Figure S4: Effects of Intra-VMN siRNA Administration on Aromatase Protein Expression in other Hypothalamic Metabolic Regulatory Structures.

## Abbreviations

AMPK	5′-AMP-activated protein kinase
ER	estrogen receptor
ERα	ER-alpha
ERβ	ER-beta
GAD	glutamate decarboxylase_65/67_
GP	glycogen phosphorylase
GPbb	GP-brain type
GPmm	GP-muscle type
INS	insulin
IIH	insulin-induced hypoglycemia
nNOS	neuronal nitric oxide synthase
NO	nitric oxide
VMN	ventromedial hypothalamic nucleus

## References

[1] Rudolph L.M., Cornil C.A., Mittelman-Smith M.A., Rainville J.R., Remage-Healey L., Sinchak K., Micevych P.E. (2016). Actions of Steroids: New Neurotransmitters. J. Neurosci..

[2] Hewitt S.C., Korach K.S. (2018). Estrogen Receptors: New Directions in the New Millennium. Endocr. Rev..

[3] Lauber M.E., Lichtensteiger W. (1994). Pre- and postnatal ontogeny of aromatase cytochrome P450 messenger ribonucleic acid expression in the male rat brain studied by in situ hybridization. Endocrinology.

[4] Foidart A., Harada N., Balthazart J. (1995). Aromatase-immunoreactive cells are present in mouse brain areas that are known to express high levels of aromatase activity. Cell Tissue Res..

[5] Roselli C.E., Klosterman S.A., Fasasi T.A. (1996). Sex differences in androgen responsiveness in the rat brain: Regional differences in the induction of aromatase activity. Neuroendocrinology.

[6] Roselli C.E., Klosterman S.A. (1998). Sexual differentiation of aromatase activity in the rat brain: Effects of perinatal steroid exposure. Endocrinology.

[7] Roselli C.E., Abdelgadir S.E., Rønnekleiv O.K., Klosterman S.A. (1998). Anatomic distribution and regulation of aromatase gene expression in the rat brain. Biol. Reprod..

[8] Veney S.L., Rissman E.F. (2000). Immunolocalization of androgen receptors and aromatase enzyme in the adult musk shrew brain. Neuroendocrinology.

[9] Beck L.A., Wade J. (2009). Sexually dimorphic estrogen receptor alpha mRNA expression in the preoptic area and ventromedial hypothalamus of green anole lizards. Horm. Behav..

[10] Dickens M.J., Cornil C.A., Balthazart J. (2011). Acute stress differentially affects aromatase activity in specific brain nuclei of adult male and female quail. Endocrinology.

[11] Ubuka T., Tsutsui K. (2015). Review: Neuroestrogen regulation of socio-sexual behavior of males. Front. Neurosci..

[12] Cornil C.A. (2018). On the role of brain aromatase in females—Why are estrogens produced locally when they are available systemically?. J. Comp. Physiol. A Neuroethol. Sens. Neural Behav. Physiol..

[13] Balthazart J., Cornil C.A., Taziaux M., Charlier T.D., Baillien M., Ball G.F. (2006). Rapid changes in production and behavioral action of estrogens. Neuroscience.

[14] Krause W.C., Ingraham H.A. (2017). Origins and functions of the ventrolateral VMH: A complex neuronal cluster orchestrating sex differences in metabolism and behavior. Adv. Exp. Med. Biol..

[15] Cryer P.E. (2013). Hypoglycemia-associated autonomic failure in diabetes. Handb. Clin. Neurol..

[16] Cryer P.E. (2014). Glycemic goals in diabetes: Trade-off between glycemic control and iatrogenic hypoglycemia. Diabetes.

[17] Ribeiro A.C., LeSauter J., Dupre C., Pfaff D.W. (2009). Relationship of arousal to circadian anticipatory behavior: Ventromedial hypothalamus: One node in a hunger-arousal network. Eur. J. Neurosci..

[18] Micevych P.E., Meisel R.L. (2017). Integrating neural circuits controlling female sexual behavior. Front. Syst. Neurosci..

[19] Shimazu T., Minokoshi T. (2017). Systemic glucoregulation by glucose-sensing neurons in the ventromedial hypothalamic nucleus (VMN). J. Endocr. Soc..

[20] Liu H., Xu Y., Hu F. (2020). AMPK in the ventromedial nucleus of the hypothalamus: A key regulatory for thermogenesis. Front. Endocrinol..

[21] Henderson L.A., Macefield V.G. (2021). The role of the dorsomedial and ventromedial hypothalamus in regulating behaviorally coupled and resting autonomic drive. Handb. Clin. Neurol..

[22] Watts A.G., Donovan C.M. (2010). Sweet talk in the brain: Glucosensing, neural networks, and hypoglycemic counterregulation. Front. Neuroendocrinology.

[23] Donovan C.M., Watts A.G. (2014). Peripheral and central glucose sensing in hypoglycemic detection. Physiology.

[24] Oomura Y., Ono H., Ooyama H., Wayner M.J. (1969). Glucose and osmosensitive neurons of the rat hypothalamus. Nature.

[25] Ashford M.L.J., Boden P.R., Treherne J.M. (1990). Glucose-induced excitation of hypothalamic neurons is mediated by ATP-sensitive K+ channels. Pfugers Arch..

[26] Silver I.A., Erecińska M. (1998). Glucose-induced intracellular ion changes in sugar-sensitive hypothalamic neurons. J. Neurophysiol..

[27] Borg M.A., Sherwin R.S., Borg W.P., Tamborlane W.V., Shulman G.I. (1997). Local ventromedial hypothalamus glucose perfusion blocks counterregulation during systemic hypoglycemia in awake rats. J. Clin. Investig..

[28] Borg M.A., Tamborlane W.V., Shulman G.I., Sherwin R.S. (2003). Local lactate perfusion of the ventromedial hypothalamus suppresses hypoglycemic counterregulation. Diabetes.

[29] Chan O., Zhu W., Ding Y., McCrimmon R.J., Sherwin R.S. (2006). Blockade of GABA(A) receptors in the ventromedial hypothalamus further stimulates glucagon and sympathoadrenal but not the hypothalamo-pituitary-adrenal response to hypoglycemia. Diabetes.

[30] Fioramonti X., Marsollier N., Song Z., Fakira K.A., Patel R.M., Brown S., Duparc T., Pica-Mendez A., Sanders N.M., Knauf C. (2010). Ventromedial hypothalamic nitric oxide production is necessary for hypoglycemia detection and counterregulation. Diabetes.

[31] Routh V.H., Hao L., Santiago A.M., Sheng Z., Zhou C. (2014). Hypothalamic glucose sensing: Making ends meet. Front. Syst. Neurosci..

[32] Ibrahim M.M.H., Bheemanapally K., Alhamami H.N., Briski K.P. (2020). Effects of intracerebroventricular glycogen phosphorylase inhibitor CP-316,819 infusion on hypothalamic glycogen content and metabolic neuron AMPK activity and neurotransmitter expression in the male rat. J. Mol. Neurosci..

[33] Ali M.H., Alshamrani A.A., Napit P.R., Briski K.P. (2022). Single-cell multiplex qPCR evidence for sex-dimorphic glutamate decarboxylase, estrogen receptor, and 5′-AMP-activated protein kinase alpha subunit mRNA expression by ventromedial hypothalamic nucleus GABAergic neurons. J. Chem. Neuroanat..

[34] Mahmood A.S.M.H., Uddin M.M., Mandal S.K., Ibrahim M.M.H., Alhamami H.N., Briski K.P. (2018). Sex differences in forebrain estrogen receptor regulation of hypoglycemic patterns of counter-regulatory hormone secretion and ventromedial hypothalamic nucleus gluco-regulatory neurotransmitter and astrocyte glycogen metabolic enzyme expression. Neuropeptides.

[35] Uddin M.M., Bheemanapally K., Ibrahim M.M.H., Briski K.P. (2020). Sex-dimorphic neuroestradiol regulation of ventromedial hypothalamic nucleus glucoregulatory transmitter and glycogen metabolism enzyme protein expression in the rat. BMC Neurosci..

[36] Stobart J.L., Anderson C.M. (2013). Role of astrocytes as gatekeepers of neuronal energy supply. Front. Cell. Neurosci..

[37] Laming P.R., Kimelberg H., Robinson S., Salm A., Hawrylak N., Müller C., Roots B., Ng K. (2000). Neuronal-glial interactions and behaviour. Neurosci. Biobehav. Rev..

[38] Gruetter R. (2003). Glycogen: The forgotten cerebral energy store. J. Neurosci. Res..

[39] Brown A.M. (2004). Brain glycogen re-awakened. J. Neurochem..

[40] Bélanger M., Allaman I., Magistretti P.J. (2011). Brain energy metabolism: Focus on astrocyte-neuron metabolic cooperation. Cell Metab..

[41] Nadeau O.W., Fontes j.D., Carlson G.M. (2018). The regulation of glycogenolysis in the brain. J. Biol. Chem..

[42] Müller M.S., Pedersen S.E., Walls A.B., Waagepetersen H.S., Bak L.K. (2015). Isoform-selective regulation of glycogen phosphorylase by energy deprivation and phosphorylation in astrocytes. Glia.

[43] Alhamami H.N., Alshamrani A., Briski K.P. (2017). Inhibition of glycogen phosphorylase stimulates ventromedial hypothalamic nucleus AMP-activated protein kinase: Activity and neuronal nitric oxide synthase protein expression in male rats. Physiol. Rep..

[44] Bheemanapally K., Ibrahim M.M.H., Briski K.P. (2020). Combinatory high-resolution microdissection/ultra-performance liquid chromatographic-mass spectrometry approach for small tissue volume analysis of rat brain glycogen. J. Pharmaceut. Biomed. Anal..

[45] Gilda J.E., Gomes A.V. (2015). Western blotting using in-gel protein labeling as a normalization control: Stain-free technology. Methods Mol. Biol..

[46] Moritz C.P. (2017). Tubulin or not tubulin: Heading toward total protein staining as loading control in Western blots. Proteomics.

[47] Bheemanapally K., Ibrahim M.M., Briski K.P. (2020). Ultra-high-performance liquid chromatography-electrospray ionization-mass spectrometry for high-neuroanatomical resolution quantification of brain estradiol concentrations. J. Pharmaceut. Biomed. Anal..

[48] Uddin M.M., Mahmood A.S.M.H., Ibrahim M.M.H., Briski K.P. (2019). Sex dimorphic estrogen receptor regulation of ventromedial hypothalamic nucleus glucoregulatory neuron adrenergic receptor expression in hypoglycemic male and female rats. Brain Res..

[49] Ibrahim M.M.H., Alhamami H.N., Briski K.P. (2019). Norepinephrine regulation of ventromedial hypothalamic nucleus metabolic transmitter biomarker and astrocyte enzyme and receptor expression: Impact of 5′ AMP-activated protein kinase. Brain Res..

[50] Zhang Q.G., Wang R., Tang H., Dong Y., Chan A., Sareddy G.R., Vadlamadui R.K., Brann D.W. (2014). Brain-derived estrogen exerts anti-inflammatory and neuroprotective actions in the rat hippocampus. Mol. Cell. Endocrinol..

[51] Oride A., Kanasaki H., Tumurbaatar T., Zolzaya T., Okada H., Hara T., Kyo S. (2020). Effects of the Fertility Drugs Clomiphene Citrate and Letrozole on Kiss-1 Expression in Hypothalamic Kiss-1-Expressing Cell Models. Reprod. Sci..

[52] Cornil C.A., Ball G.F., Balthazart J. (2020). Sexually differentiated and neuroanatomically specific co-expression of aromatase neurons and GAD67 in the male and female quail brain. Eur. J. Neurosci..

