# Supplementary material for: Effects of Ventromedial Hypothalamic Nucleus (VMN) Aromatase Gene Knockdown on VMN Glycogen Metabolism and Glucoregulatory Neurotransmission
Source: Biology (Basel). 2023 Feb 3;12(2):242. doi: 10.3390/biology12020242 (PMC9953379; doi:10.3390/biology12020242)
Supplement: Supplementary file 1 [file biology-12-00242-s001.zip › biology-2186472-supplementary.pdf]

Supplementary Figure S1 Representative Rostral VMN Target Protein Full-Length Western Blots

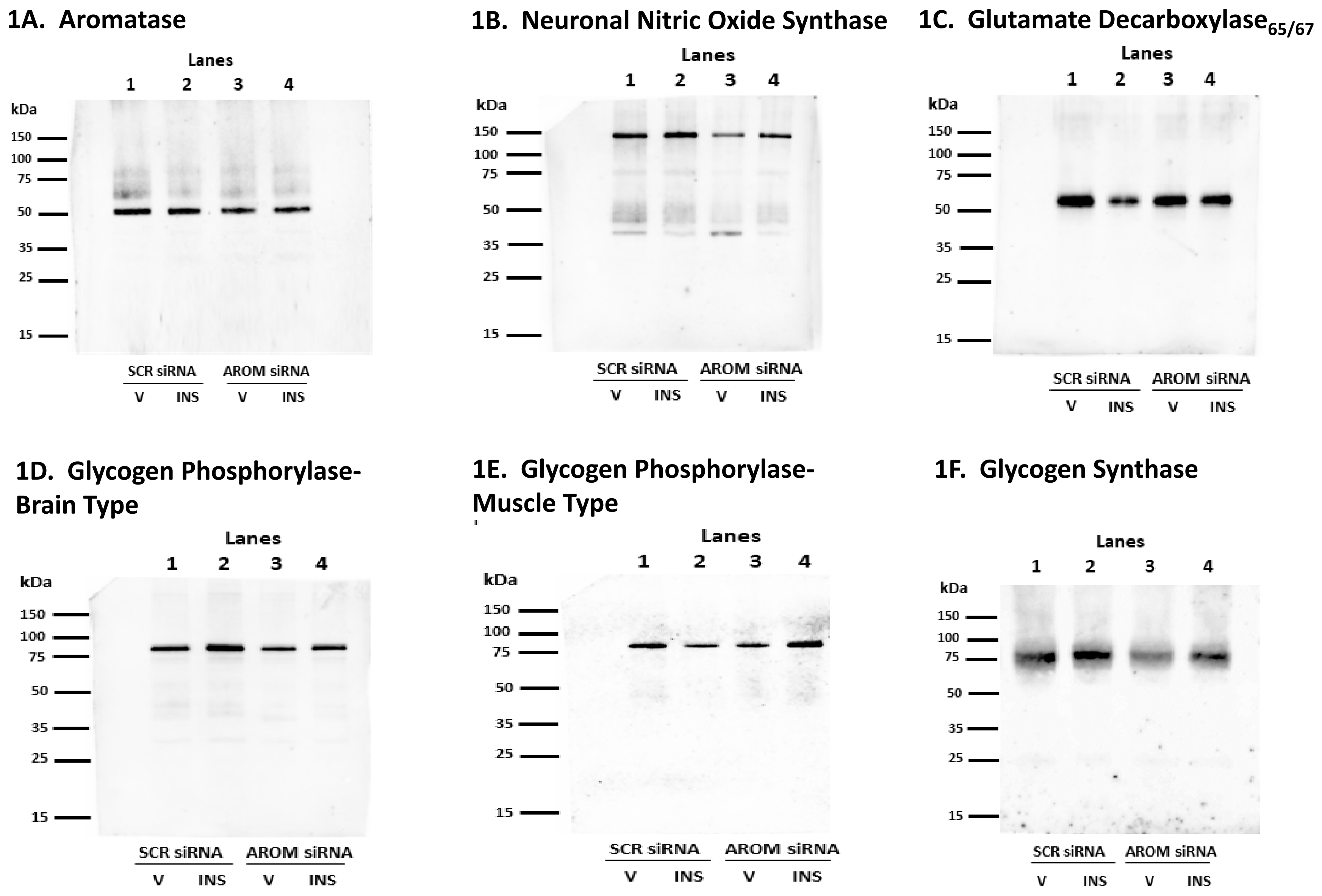

Figure S1 Legend: Figures 1A-1F depict full-length, uncropped immunoblots for rostral (-1.8 to -2.3 mm posterior to bregma) ventromedial hypothalamic nucleus (VMN) micropunch-dissected tissue sample aromatase (AROM; 1A), neuronal nitric oxide synthase (1B), glutamate decarboxylase$_{65/67}$ (1C), glycogen phosphorylase-brain type (1D), glycogen phosphorylase-muscle type (1E), or glycogen synthase (1F) protein levels in groups of male rats pretreated by intra-VMN administration of scramble (SCR) or AROM siRNA prior to subcutaneous injection of vehicle (V) or insulin (I). Molecular weight markers (15-150 kDa) are depicted to the right of each blot.

Supplementary Figure S2 Representative Middle VMN Target Protein Full-Length Western Blots

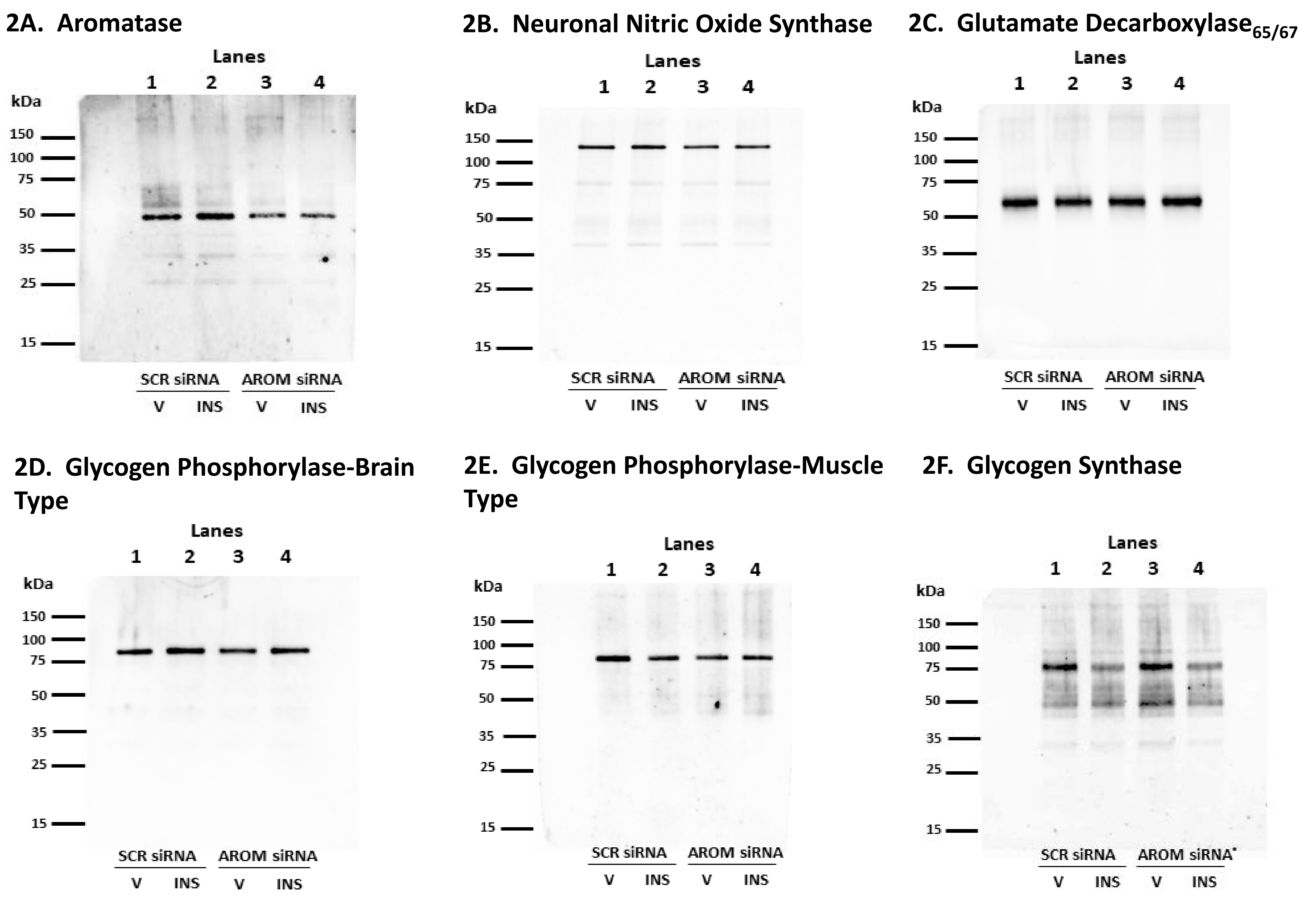

Figure S2 Legend: Figures 2A-1F depict full-length, uncropped immunoblots for middle VMN (-2.3 to -2.8 mm posterior to bregma) micropunch-dissected tissue sample AROM (2A), neuronal nitric oxide synthase (2B), glutamate decarboxylase$_{65/67}$ (2C), glycogen phosphorylase-brain type (2D), glycogen phosphorylase-muscle type (2E), or glycogen synthase (2F) protein levels in groups of male rats pretreated by intra-VMN administration of 0 SCR or AROM siRNA prior to subcutaneous injection of V or 0 I. Molecular weight markers (15-150 kDa) are depicted to the right of each blot.

Supplementary Figure S3 Representative Caudal VMN Target Protein Full-Length Western Blots

3A. Aromatase

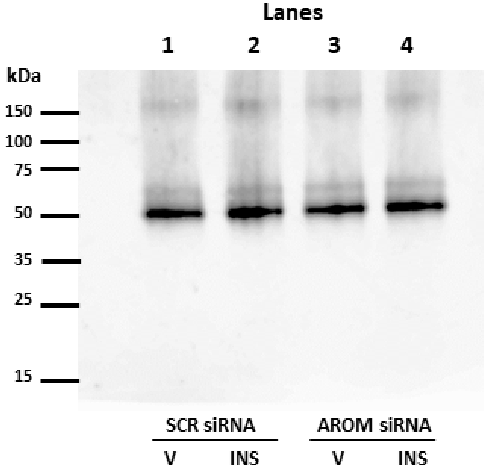

3B. Neuronal Nitric Oxide Synthase

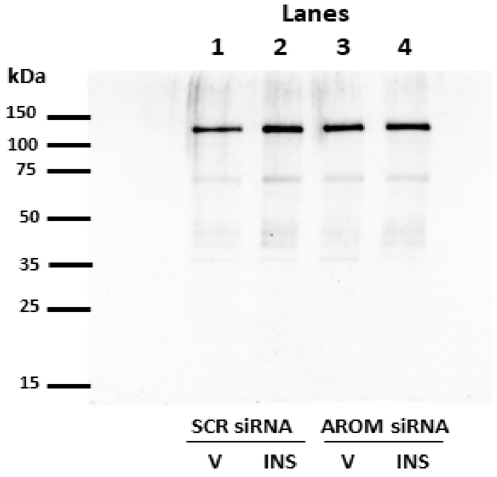

3C. Glutamate Decarboxylase$_{65/67}$

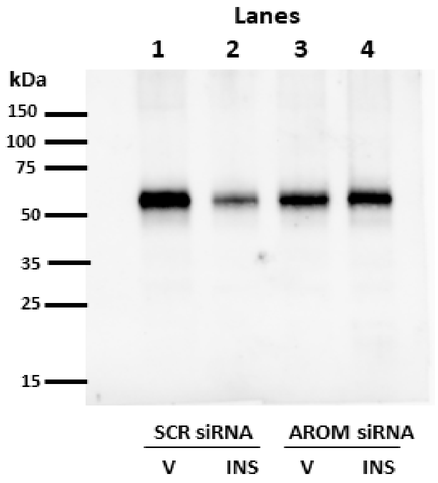

3D. Glycogen Phosphorylase-Brain Type

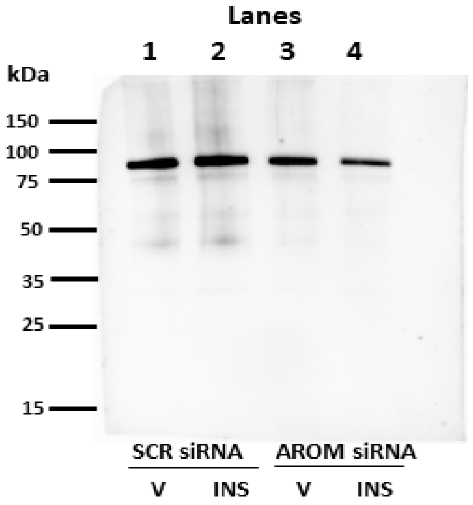

3E. Glycogen Phosphorylase-Muscle Type

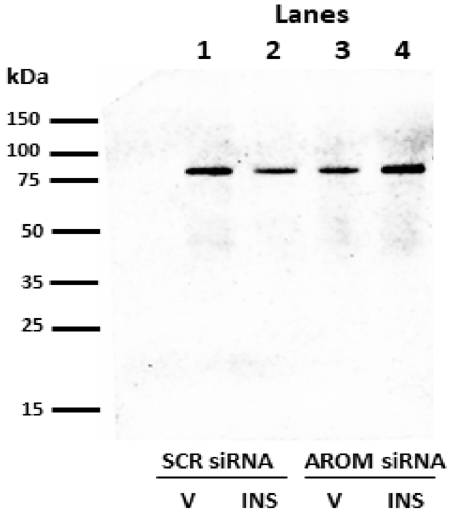

3F. Glycogen Synthase

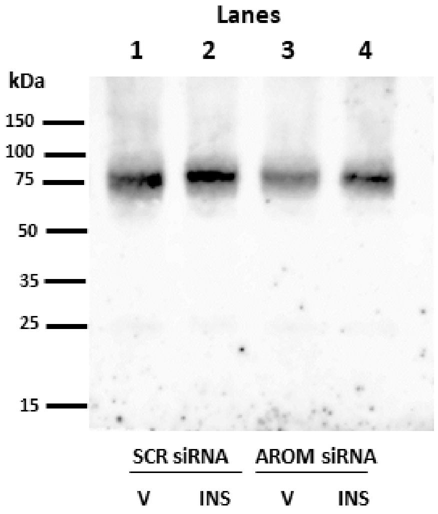

Figure S3 Legend: Figures 3A-1F depict full-length, uncropped immunoblots for caudal VMN (-2.3 to -2.8 mm posterior to bregma) micropunch-dissected tissue sample AROM (3A), neuronal nitric oxide synthase (3B), glutamate decarboxylase$_{65/67}$ (3C), glycogen phosphorylase-brain type (3D), glycogen phosphorylase-muscle type (3E), or glycogen synthase (3F) protein levels in groups of male rats pretreated by intra-VMN administration of SCR or AROM siRNA prior to subcutaneous injection of V or I. Molecular weight markers (15-150 kDa) are depicted to the right of each blot.

Supplementary Figure S4 Effects of Intra-VMN siRNA Administration on Aromatase Protein Expression in other Hypothalamic Metabolic Regulatory Structures

4A. PVN Aromatase

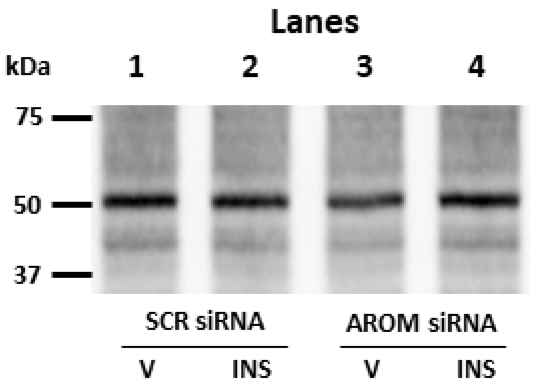

4B. DMN Aromatase

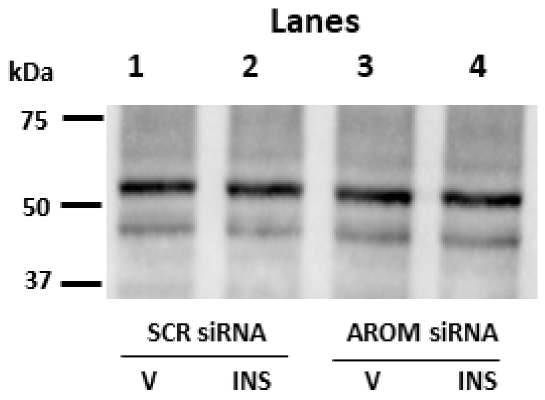

4C. LHA Aromatase

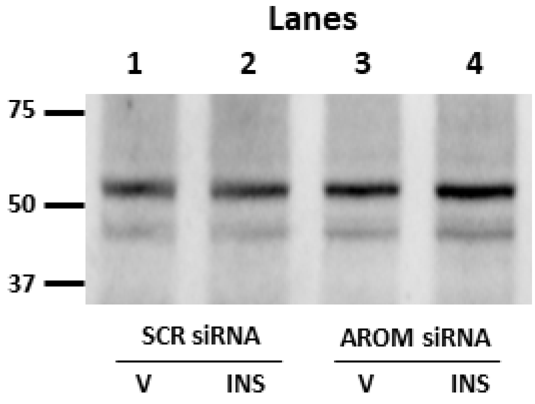

4D. ARH Aromatase

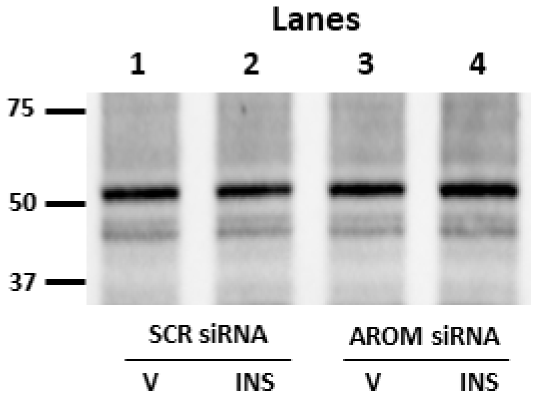

Figure S4 Legend: Figures 4A-4D show that aromatase protein expression in the PVN, DMN, LHA, and ARH, respectively, is unaffected in each location by siRNA-mediated VMN aromatase gene knockdown.
